# Covid-19 pandemic and mental health among adolescents and young adults: results from two studies conducted in North of Italy

**DOI:** 10.1192/j.eurpsy.2024.683

**Published:** 2024-08-27

**Authors:** L. Pedrini, S. Meloni

**Affiliations:** ^1^UO Psichiatria, IRCCS Centro San Giovanni di Dio, Fatebenefratelli di Brescia, Brescia, Italy

## Abstract

**Introduction:**

The northern region of Italy had been the epicenter of the first wave of Covid-19. The youth population residing in this area experienced an extended period of restrictive measures implemented to curb the spread of the virus. Given the adverse effects of social distancing and lockdown measures, there is a concern regarding the mental health of young individuals in this region. However, there remains a notable scarcity of studies exploring the long-term impact of the pandemic on the mental health of this vulnerable population.

**Objectives:**

To assess psychopathological symptoms among adolescents and young adults in order to evaluate the extent of their impact in the context of the Covid-19 pandemic. To explore potential risk factors and resilience factors in youth who have experienced the effects of the pandemic.

**Methods:**

Two studies are performed. *Study n1* has observational design and includes 7,146 adolescents and young adults (age range 14–25) evaluated during the fourth wave of the COVID-19 through standardized measures for depression, anxiety, anger, somatic symptoms, resilience, loneliness and post-traumatic growth. *Study n2* has prospective design and includes 153 students (mean age 16.1±0.49), evaluated before the Covid-19 pandemic (November 2019–January 2020) and 1 year later (April–May 2021) to measure anxiety, depression, stress, emotional dysregulation, maladaptive behaviours.

**Results:**

Study 1. Clustering methods identifed two groups of students with different psychological features, that we further defined as “poor mental health” and “good mental health”. Those with poor mental health were characterized by higher scores of loneliness and self-harm, followed by being of female gender, presenting binge eating behaviors and, finally, having unsatisfying family relationships.

Study 2. Over the course of one year, significant changes in various psychological parameters were observed: an increase in anxiety, stress for future uncertainty, and higher frequency of maladaptive behaviours.Stress related to social domains (i.e., school attendance, romantic relationships, peer pressure) decreased over the year. Cluster analysis identified three distinct groups of youths based on their changes in psychopathological symptoms over time: those who worsened (N=23; 15%), improved (N=55; 34%), or remained stable (N=75; 46%). Furthermore, adolescents who reported an increase in self-harm (OR=2.61; p<0.001), binge-drinking (OR=3.0; p=0.007), aggressiveness (OR 1.92; p=0.004), and binge-eating (OR 2.55; p=0.003) were more likely to be associated with a worsened mental health condition.

**Conclusions:**

The findings from these studies substantiated the significant psychological distress caused by the COVID-19 pandemic. Furthermore, they yielded valuable further insights regarding into the factors linked to distinct patterns of mental health outcomes.

**Disclosure of Interest:**

None Declared

